# Self-Assembly of Discrete Metallocycles *versus* Coordination Polymers Based on Cu(I) and Ag(I) Ions and Flexible Ligands: Structural Diversification and Luminescent Properties

**DOI:** 10.3390/polym8020046

**Published:** 2016-02-15

**Authors:** Javier Vallejos, Iván Brito, Alejandro Cárdenas, Michael Bolte, Sergio Conejeros, Pere Alemany, Jaime Llanos

**Affiliations:** 1 Departamento de Química, Universidad Católica del Norte, Av. Angamos 0610, Antofagasta 124000, Chile; jvallejos@ucn.cl (J.V.); Jllanos@ucn.cl (J.L.); 2 Departamento de Química, Universidad de Antofagasta, Av. Angamos 601, Antofagasta 124000, Chile; 3 Departamento de Física, Universidad de Antofagasta, Av. Angamos 601, Antofagasta 124000, Chile; alejandro.cardenas@uantof.cl; 4 Institut für Anorganische Chemie der Goethe-Universität Frankfurt, Max-von-Laue-Strasse 7, D-60438 Frankfurt am Main, Germany; bolte@chemie.uni-frankfurt.de; 5 Departament de Química Física and Institut de Química Teòrica i Computacional (IQTCUB), Universitat de Barcelona, Martí i Franqués 1, Barcelona 08028, Spain; S.Conejeros@bristol.ac.uk (S.C.); p.alemany@ub.edu (P.A.)

**Keywords:** metal-organic framework, luminescence, coordination polymer, MOF

## Abstract

Three new Ag(I) and one Cu(I) coordination compounds with two different positional isomers, propane-1,3-diyl bis(pyridine-4-carboxylate) (L1) and propane-1,3-diyl bis(pyridine-3-carboxylate) (L2), of a bis-(pyridyl-carboxylate) ligand have been synthesized. X-ray diffraction analysis revealed that the self-assembly of L1 with AgCF_3_SO_3_ and AgClO_4_ salts leads to the formation of discrete binuclear metallocycles {Ag(L1)CF_3_SO_3_}_2_ (**1**) and {Ag(L1)ClO_4_}_2_ (**2**), respectively. However, self-assembly of the other ligand, L2, with AgCF_3_SO_3_ and CuCl salts, results in a 1-D zig-zag chain {Ag(L2)CF_3_SO_3_}_∞_ (**3**) and a 1-D double-stranded helical chain {Cu_2_Cl_2_(L2)_2_}_∞_ (**4**) coordination polymers, respectively. Solid emission spectra recorded at room temperature show interesting luminescence properties for all four compounds in the range from 438 to 550 nm, especially for compound **4** that was found to change its emission color when the wavelength of the excitation radiation is switched from 332 to 436 nm.

## 1. Introduction

Self-assembly of coordination polymers and metal-organic frameworks (MOFs) [1,2,3] has been attracting great attention in the last decade, mainly because of their great potential as functional materials for diverse technological applications [4,5,6,7,8]. In particular, the luminescent properties of this type of materials and the possibility of fine-tuning the characteristics of their emission by carefully selecting both the metal and the organic ligands have been a topic of special relevance in this field [9,10,11,12,13]. In this sense, the well-studied luminescent properties [14] of d^10^cations, such as Cu(I), Ag(I), and Au(I), as well as their versatility in the construction of complex coordination networks with different types of organic ligands, have been the object of interest.

From the structural point of view, the two principal themes in this field have been the synthesis of compounds having either discrete molecular architectures with polyhedral or polygonal shapes or infinite coordination polymers in one-, two-, or three-dimensions (1-D, 2-D, or 3-D) of metal ions in combination with deliberately-tailored organic ligands. In the latter case, the resulting network topology [15] for the supramolecular complex can be usually more or less predicted by selecting the chemical structure of the organic ligands and the usual coordination geometry of the metal ions linking them together in the final structure [16]. In this sense, structural aspects of the bridging ligands, such as their rigidity or flexibility, length, size, bulkiness, linear, or nonlinear geometry, have been found to play a crucial role in the construction of specific architectures [17,18,19].

In the past few years, extensive studies have been carried out using rigid bridging ligands such as 4,4’-bipyridine-type compounds [20,21,22,23], Schiff-bases [24,25,26,27,28], or 2,4,6-tri(4-pyridyl)-1,3,5-triazine [29,30,31,32], in combination with silver to obtain Ag(I) polymeric networks with beautiful topologies and inclusion behaviors [33,34,35]. By contrast, the use of flexible ligands with several degrees of freedom and, hence, few conformational restraints have been avoided basically due to the unpredictable nature of the structure for the resulting polymers [36,37], since in order for such complexes to be potentially useful it is essential that their structures can be predictably tuned via variations in their constituent building blocks. In this respect, the rational design of coordination polymer architectures using flexible ligands is still a challenge. On the other hand, it has been observed that when flexible bipyridyl ligands adopting different conformations react with silver salts, new interesting and unusual ligand structures may facilitate the formation of helixes and other novel supramolecular architectures that are geometrically impossible to obtain with rigid linkers [38,39,40,41].

For a large number of silver (I) complexes reported in the literature, their photophysical properties remain largely unexplored, a situation that is generally attributed to the difficulty of performing experiments with these compounds due to the high photosensitivity exhibited by many compounds containing silver. Room-temperature phosphorescence has been reported for just a few Ag(I) compounds [38,39,40,41,42,43,44], while, in contrast, related multinuclear compounds with Cu(I) or Au(I) ions have attracted a considerable interest due to their unusual structural and photoluminescence properties [45,46]. In particular, cuprous compounds with halogen and pyridine-based ligands provide remarkable structural diversity depending on their stoichiometry. Diverse systems based on Cu(I), ranging from mononuclear coordination compounds to stair-step polymers, have been prepared from the simple combination of cuprous halide with pyridine-based ligands [47]. Since the d^10^ electronic configuration of Cu(I) enforces no special stereochemical demands, the coordination sphere is largely determined by electrostatic and geometric factors. Moreover, crystalline solids isolated from solutions may be in either tetranuclear or polymeric forms, depending on the crystallization conditions. As it will be shown below, the structural ambiguity found for these systems provides an additional challenge to the characterization of their photophysical properties.

**Scheme 1:**
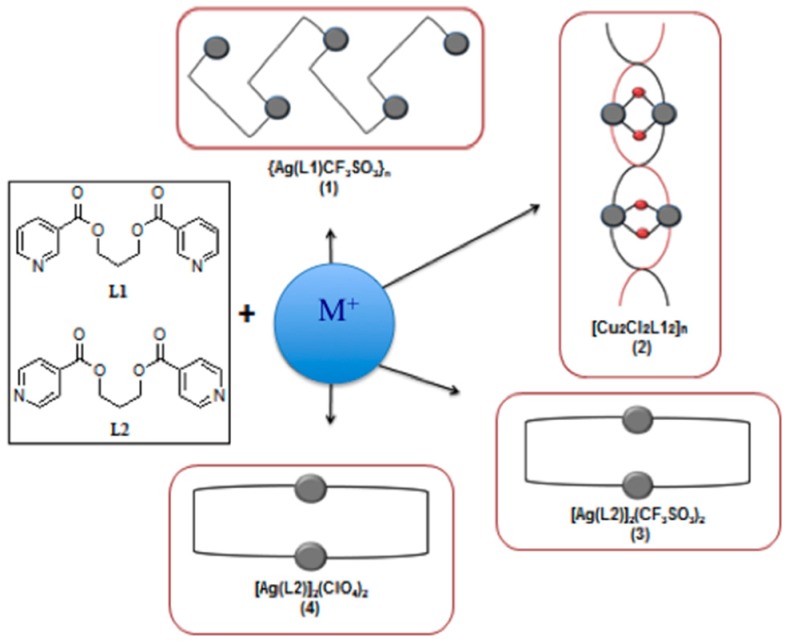
Schematic description of the compounds presented in this work. M^+^ = Cu(I), Ag(I).

**Scheme 2:**
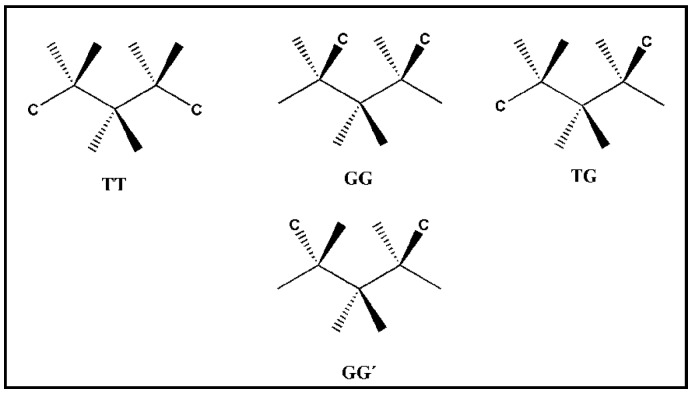
The four possible conformations for the propyl backbone of the L1 and L2 ligands where C is a functional group, nicotinate, or isonicotinate in our case.

This work forms part of our continuing efforts in the synthesis, structural characterization, and determination of the photophysical properties of metal-organic hybrids based on d^10^ ions and flexible organic ligands [40,41,48,49,50]. In this report, we have selected propane-1,3-diyl bis-(pyridine-4-carboxylate), L1, and propane-1,3-diyl bis-(pyridine-3-carboxylate), L2, two positional isomers, as the tecton (Scheme 1) [48,49,50]. These two ligands have a non-rigid propyl spacer group (–CH_2_–CH_2_–CH_2_–) that plays a decisive role in providing a rich set of conformational isomers for each of the two ligands. As shown in Scheme 2, if we take only into account the flexibility of the propyl linker, L1 and L2 can exist, at least, in four different conformations, labeled as TT, TG, GG, and GG’ (where T stands for trans and G for gauche) [38,39,40,41,43,44] which when combined with d^10^ ions may afford different supramolecular assemblies leading to diverse photophysical properties. In this study we report the synthesis, the crystal structures, and the luminescent properties of four such d^10^ complexes formed with the isomeric bis-(pyridyl-carboxylate) ligands L1 and L2 and different metals or counteranions: {Ag(L1)CF_3_SO_3_}_2_ (**1**), {Ag(L1)ClO_4_}_2_ (**2**), {Ag(L2)CF_3_SO_3_}_∞_ (**3**), and {Cu_2_Cl_2_(L2)_2_}_∞_ (**4**).

## 2. Experimental Section

### 2.1. Coordination Polymer Synthesis and Characterization

All chemicals were of A.R. grade and used without further purification. The organic ligands L1 and L2 were prepared according to standard methods reported in the literature [48,49,50]. FTIR spectra in the range 400–4000 cm^−1^ were obtained, using KBr pellets, with a Nicolet Avatar 330 spectrometer (Thermo Scientific, Waltman, MA, USA)

For the synthesis of the compounds **1** and **3** we used a solution of AgCF_3_SO_3_ (25.6 mg, 0.1 mmol) in water which was slowly added to a solution of the L1 or L2 ligands (27.2 mg, 0.1 mmol) in THF (4 mL) respectively, (yields 82% and 52% for **1** and **3**, respectively). For the synthesis of compound **2** we used a solution of AgClO_4_ (19.1 mg, 0.1 mmol) in water which was slowly added to a solution of the L1 ligand (27.2 mg, 0.1 mmol) in THF (4 mL) (yield 38%). Colorless single crystals suitable for X-ray analysis were obtained after a few days for **1**, **2**, and **3**. For the synthesis of compound **4** we used a solution of CuCl (9.9 mg, 0.1 mmol) in water which was slowly added to a solution of the L2 ligand (27.2 mg, 0.1 mmol) in THF (4 mL) (yield 43%). Brown-yellow single crystals suitable for X-ray analysis were obtained after a few days.

The UV–Vis spectra were recorded on a Perkin Elmer Lambda 20 spectrophotometer (Perkin-Elmer, Waltman, MA, USA) with diffuse reflectance sphere in the range of 200–400 nm in the solid state at room temperature. The photoluminescence (PL) spectra (emission) were measured using the JASCO FP-6500 spectrofluorometer (Jasco Corporation, Tokyo, Japan). All spectra were recorded at room temperature. In order to compare photoluminescence intensity, sample weight was the same in all experiments.

### 2.2. X-ray Crystallographic Measurements

Single crystal analysis were performed at 173(2) K with a STOE IPDS II (two circle diffractometer with Mo *K*α radiation (λ = 0.71073 Å) by the ω-2θ scan technique STOE&Cie (Darmstadt, Germany). All data were collected for absorption by a multi-scan method [51]. The program X-Area was applied for integration of the diffraction profiles. The structures were solved by direct methods using SHELXS-90 [52], followed by structure refinement on F^2^ with program SHELXL-2014/6 [53]. All non-hydrogen atoms were refined anisotropically. The hydrogen atoms were geometrically positioned with isotropic thermal parameters set to 1.2 U_eq_ of the parent atom. Crystal data for compounds **1**–**4** are given in Table 1. The CCDC reference numbers for the four new compounds are: 1033654, 1033655, 1033652, and 1033653. The phase purity of all four newly-synthetized compounds was verified by powder X-ray diffraction (PXRD) determination (see supplementary material Figures S1–S4).

## 3. Results and Discussion

### 3.1. Conformational Flexibility of the Organic Linkers

**Scheme 3:**
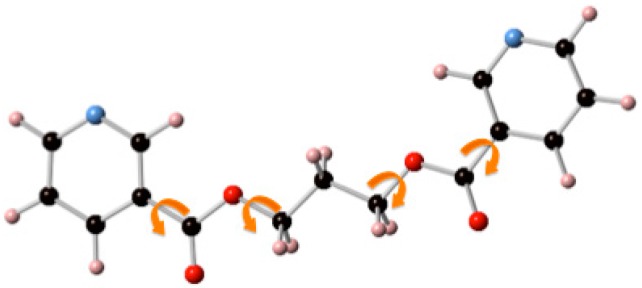
Internal rotations allowing the existence of several conformers within each of the four GG, GG’, GT, and TT families of conformers for ligands L1 (not shown here) and L2.

As mentioned above, in this work we have used two flexible ligands, L1 and L2, in which two pyridine rings are linked by a non-rigid propyl spacer group (Scheme 1). Considering the disposition of the two pyridine rings with respect to the bridging –CH_2_–CH_2_–CH_2_– chain we can distinguish four different groups of conformers: GG, GG’, GT, and TT, as shown in Scheme 2. Additional rotational freedom around the C–O bonds linking the carboxylate group to the spacer or around the C–C bond between the ring and the carboxylate group (Scheme 3) allows for the existence of several conformers within each of the four GG, GG’, GT, and GT sets of conformers.

**Figure 1:**
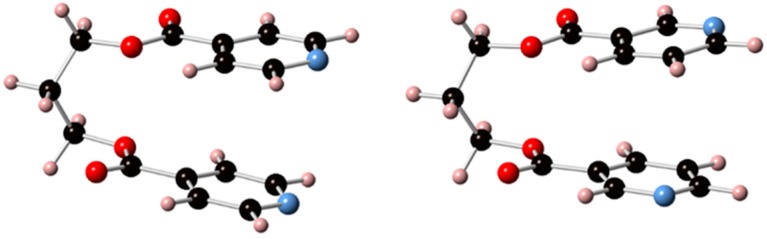
Molecular geometry for the most stable conformers of ligands L1 (**left**) and L2 (**right**).

Relative energies for the different conformers of L1 and L2 in vacuum were obtained by optimization of the molecular geometry for each conformer using the density functional theory-based M062X/6-31G(d,p) [54] method as implemented in the GAUSSIAN-09 suite of programs [55]. These calculations indicate that, for the two ligands, the most stable conformations are of the GG-type, with a characteristic C-shaped structure (Figure 1) in which the two rings are practically coplanar with an interplanar distance about 2.5 Å. Totally-extended TT-type conformations are highest in energy, about 6 kcal/mol above the GG ones, while the GG’ and GT-type conformations can be found in between those two, about 4–5 kcal·mol^−1^ above the most stable GG ones.

The N···N distance in the most stable conformation for L1 is 3.7 Å, just a little bit longer than the Ag···Ag distances found in coordination polymers of this metal, which are in the range 3.0–3.5 Å [56,57,58] although the flexible nature of the ligand should allow it to adapt its bite size to this range of distances without a considerable reorganization energy demand. As seen in Figure 1, in the most stable conformer for ligand L2 the two nitrogen atoms on the pyridine rings are disposed in opposite directions, a geometry that prevents the coordination of a single ligand molecule with the same Ag···Ag pair, favoring in this way the formation of extended structures over that of discrete [2 + 2] macrocyclic complexes. The situation is, however, not so simple, since the rotation of one of the aromatic fragments around the C–O bond that links it to the propyl spacer gives an alternative GG-type conformer in which the two N atoms point in the same direction with an N···N separation of 3.4 Å, which is only about 1.5 kcal·mol^−1^ higher in energy. This small energy difference seems to indicate that the observed preference (see below) of each single molecule of ligand L2 to combine with two different Ag···Ag pairs as opposed to those of ligand L1 which preferentially binds to the same Ag···Ag pair, seems to be due to kinetic, rather than thermodynamic, reasons.

### 3.2. Structural Analysis

**Figure 2:**
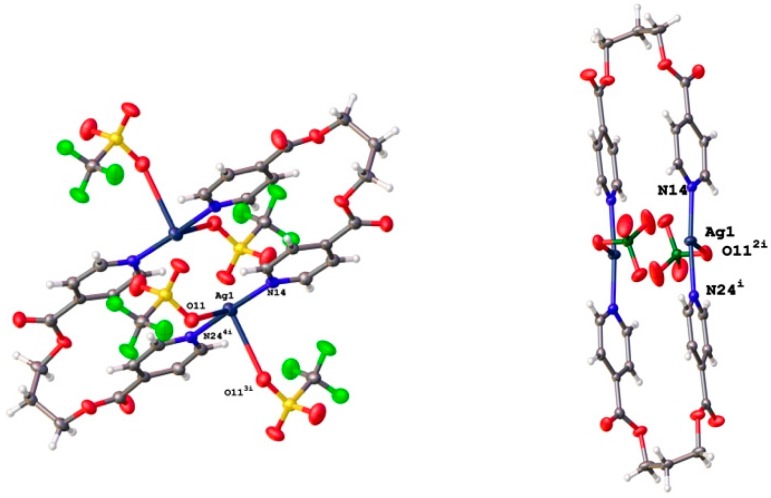
View of the metallocyclic [2 + 2] structures found for [Ag(L1)]_2_(CF_3_SO_3_)_2_, compound **1** (**left**); and [Ag(L1)]_2_(ClO_4_)_2_, compound **2** (**right**).

The formulation [Ag(L1)]_2_(CF_3_SO_3_)_2_ and [Ag(L1)]_2_(ClO_4_)_2_ for compounds (**1**) and (**2**), respectively, was confirmed by single-crystal X-ray diffraction. The X-ray structural analysis revealed for them the presence of [2 + 2] discrete macrocyclic complexes (Figure 2) in which a single Ag···Ag pair binds to two ligand molecules, thus preventing the formation of an extended structure with a skeleton formed by Ag–L bonds.

The coordination of two L1 molecules in a GG conformation to a pair of symmetry-related silver atoms results in the formation of 32-membered metallocyclic rings in which the Ag∙∙∙Ag separations are 3.5126(8) Å and 3.4288(5) Å in **1** and **2**, respectively, pointing at the presence of weak Ag∙∙∙Ag interactions in the two cases. In **1**, each Ag atom is coordinated to the two N atoms on the ligand and to an O atom of CF_3_SO_3_^−^ anion with an distance of 2.626(4)Å. In addition, there is another short Ag–O contact of 2.890(4) Å from the Ag center to a symmetry-related [Ag(L1)_2_]_2_(CF_3_SO_3_)_2_ unit. The N–Ag–N angle is 175.11(19)°, the N–Ag–O angles are 98.11(13)°, 93.07(16)°, 91.70(15)°, 80.10(12)°, 98.48(13)° and the O–Ag–O angle amounts to 98.48(13)°.

All these values are comparable to those found for other compounds with saw-horse or T-shaped coordination geometries around the Ag^+^ ion [33,59,60]. In the case of compound **2**, additional coordination to the oxygen atom on a neighboring ClO_4_^−^ anion results in a T-shaped coordination geometry for the Ag(I) ion with an N–Ag–N bond angle of 173.44(9)° and N–Ag–O angles of 87.33(7)° and 87.79(8)°. In the two compounds the two pyridyl rings coordinating to the same Ag atom are almost coplanar with torsional angles of 13.74(11)° and 3.1(2)° in **1** and **2**, respectively.

**Table 1:** Crystal data for compounds **1**–**4.**

Compound	1	2	3	4
Molecular formula	C_32_H_28_Ag_2_F_6_N_4_O_14_S_2_	C_30_H_28_Ag_2_Cl_2_N_4_O_16_	C_16_H_14_AgF_3_N_2_O_7_S	C_30_H_28_Cl_2_Cu_2_N_4_O_8_
Formula weight	1086.44	987.20	543.22	770.54
Space group	P-1	P21/c	P21/n	C2/c
a/Å	6.5913(5)	10.3551(5)	7.9745(6)	15.8963(11)
b/Å	11.7365(10)	7.5927(4)	10.9040(6)	29.2769(12)
c/Å	13.2883(10)	21.4540(9)	21.8561(13)	15.2635(10)
α/°	65.594(6)	90	90	90
β/°	89.733(6)	98.996(3)	94.865(5)	117.882(5)
γ/°	84.604(7)	90	90	90
V/Å^3^	931.30(13)	1,666.03(14)	1,893.6(2)	6,278.9(7)
*Z*	1	2	4	8
*D*_c_/Mg·m^−3^	1.937	1.968	1.905	1.630
μ/Mo*K*α/mm^−1^	1.268	1.421	1.248	1.582
*F*(000)	540	984	1080	3136
Reflections collected	15,998	25,740	11,865	52,992
Unique reflections	3,469	3,123	3,544	5,921
No. of params	271	245	271	415
GOF on *F*^2^	1.084	1.044	0.977	0.982
Final *R* indices [*I* ≥ 2σ(I)]	*R*_1_ = 0.0472, *wR*_2_ = 0.1168	*R*_1_ = 0.0274, *wR*_2_ = 0.0685	*R*_1_ = 0.0330, *wR*_2_ = 0.0848	*R*_1_ = 0.0472, *wR*_2_ = 0.1024
*R* int	0.0920	0.0620	0.0731	0.0927
Largest difference Peak, hole/e Å^−3^	0.645 and −1.537	0.771 and −0.624	0.527 and −0.998	0.650 and −0.433

**Figure 3:**
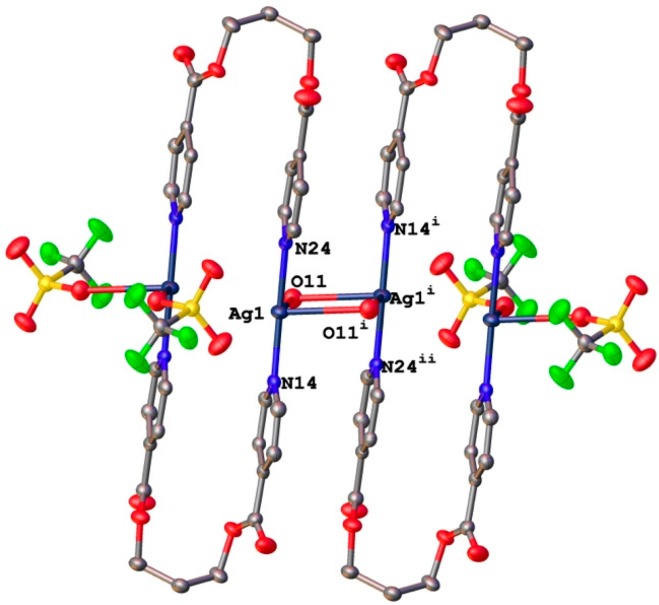
View of the stacking of discrete binuclear metallocycles along [100] in **1** arising from the coordination of Ag^+^ ions with O atoms on neighboring triflate anions. For the sake of simplicity, only one O atom from each of the triflate anions forming the central Ag_2_(μ-O)_2_ unit is shown in this representation.

As shown in Figure 3, in the crystal structure of compound **1**, neighboring metallocycles form chains along the [100] direction linked by a planar rhombic Ag_2_(μ-O)_2_ core with two symmetry-related O atoms from triflate anions.

**Figure 4:**
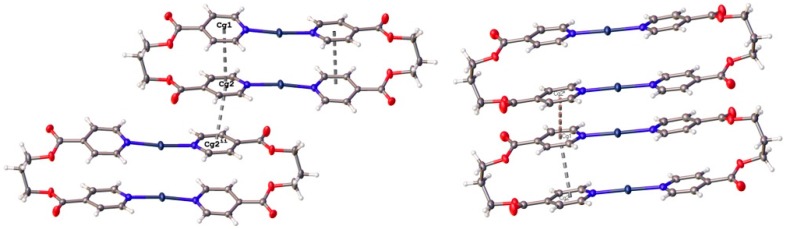
π–π stacking interactions between pyridine rings in **1** (**left**) and **2** (**right**) leading to the formation of ladder-like (**1**) or step-like (**2**) 1-D chains of (AgL1)_2_ dimers.

In the two compounds, the conformation of the metallocycles is stabilized by π–π stacking interactions between pyridine rings (Figure 4) with centroid-centroid distances of 3.710(1) and 3.679(3) Å for the intermolecular interactions in **1** and **2**, respectively. In both compounds, the binuclear units are also linked to other neighboring metallocycles by further π–π stacking interactions with centroid-centroid distances of 3.728(3) Å and 3.723(1) Å for compounds **1** and **2**, respectively.

In the two compounds there are also weak Ag···C interactions with Ag⋯C distances in the range from 3.052(3) to 3.107(2) Å that fall in the secondary bonding range (the sum of the van der Waals radii of Ag and C is 3.42 Å) [61]. These type of Ag⋯C interactions, with distances ranging between 2.80–3.34 Å, have also been reported for other polymeric silver (I) compounds [33,59,60,62] and are thought to have a decisive influence for the packing in the solid state for the compounds under study.

**Figure 5:**
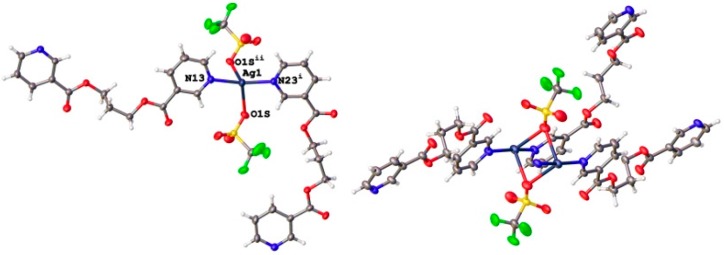
(**a**) Perspective view of the zig-zag chain in compound **3**. (**b**) View showing the links between neighboring zig-zag chains mediated by O atoms from triflate anions.

In contrast to the discrete dimeric units found when using ligand L1, compounds **3** and **4**, formed with ligand L2, have extended structures where L2 adopts a TG conformation. As shown in Figure 5a, the Ag(I) ion in **3** is tetra-coordinated, with a saw-horse coordination geometry defined by two N atoms on different L2 molecules and two O atoms from symmetry-related triflate anions with 2.171(2) and 2.172(3)° Å Ag–N distances, and 2.662(3) and 2.791(2) Å Ag–O distances. The N–Ag–N angle in this case is 166.55(9)°. The crystal structure consists of one-dimensional Ag(I)–L zigzag chains (Figure 5b), which are further linked by two O atoms from symmetry-related triflate anions forming a planar rhombic Ag_2_(μ-O)_2_ ring.

Weak Ag⋯C interactions in the secondary bonding range with Ag⋯C distances between 3.071(3) and 3.111(3) Å are also observed in this case and are believed to play an important role in the packing observed in the final crystal structure. The two pyridyl rings coordinated to a single Ag atom are also almost coplanar in **3**, with a torsional angle of 4.03(15)° between them.

In compound **4**, there are two formula units in the asymmetric unit, which are related by a pseudo-translation in the direction of the *a*-axis. This pseudo-translation is fulfilled by 91% of the atoms. If the *a*-axis is halved, the methylene chain shows disorder. Thus, it is justified to refine the structure with two symmetry independent formula units.

**Figure 6:**
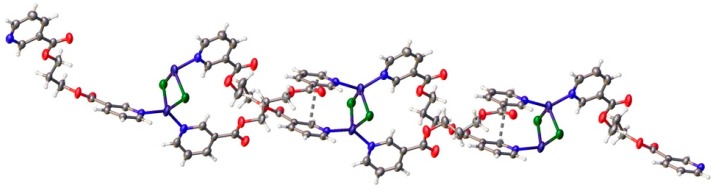
View of a double-stranded helical {Cu_2_Cl_2_(L2)_2_}_∞_ chain found in compound **4** showing the π–π stacking interactions between pyridyl rings on the two strands.

Copper (I) ions are tetra-coordinated with a distorted tetrahedral coordination environment formed by two N atoms on two different L2 molecules and two single chlorine anions (Figure 6). The Cu–N distances range from 1.973(3) to 2.002(3) Å and the Cu–Cl ones from 2.4147(11) to 2.4787(12) Å. The bond angles around Cu(I) ions are in the range from 102.20(5)° to 138.11(16)°. The relatively short Cu1···Cu1A distance, 2.982(1) Å, is indicative of weak cuprophilic interactions since in photoluminescent polynuclear copper(I) complexes that display Cu···Cu contacts below 3.0 Å the presence of these Cu^I^···Cu^I^ interactions is invariably invoked in the spectral assignment of their emissions [63,64,65,66,67,68,69,70,71,72]. In this compound, pyridyl rings coordinated to a single metal atom are no longer coplanar, with torsional angles between them of 69.05(19) and 73.7(2)°. In the crystal structure, the screw axes passing thought middle point of the planar rhombic Cu_2_(μ-Cl)_2_ cores generates one-dimensional Cu(I)-L2 double stranded helical chains reinforced by weak π–π stacking interactions between the two strands with centroid-centroid distances of 3.607(2) Å and 3.928(2) Å.

### 3.3. Luminescent Properties

**Scheme 4:**
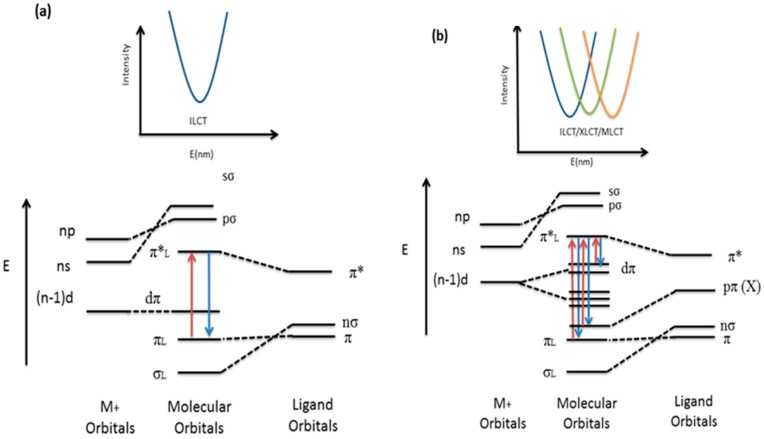
Schematic molecular orbital diagram showing the interactions of the principal MOs involved in luminescence for (**a**) d^10^ hybrid systems with electronic transitions originating mainly from the organic ligand and (**b**) d^10^ hybrid systems with electronic transitions located on the ligand, the counteranion (X), and the metal centers giving rise to different charge transfers: ILCT: Intraligand Charge Transfer, XLCT: Counteranion-Ligand Charge Transfer, MLCT: Metal-Ligand Charge Transfer.

The photophysical properties of inorganic-organic hybrid coordination compounds have been a focus of interest in recent years, mainly because of the possibility they offer to affect the emission properties (wavelength and intensity) of purely organic materials, opening the door for new potential applications as luminescent materials in devices, such as light-emitting diodes [73,74]. In this sense, the judicious choice of a combination of suitable conjugated organic linkers and transition-metal centers are believed to offer an efficient method for obtaining new types of photoluminescent materials, especially for systems containing d^10^ ions [75,76]. While luminescence in hybrid sytems with group 12 metals (Zn^2+^, Cd^2+^ and Hg^2+^) has mostly its origin in electronic transitions associated with the organic ligands (Scheme 4a), preventing the possibility of a fine tuning based on changes in the metal [70], in the case of compounds with Group 11 metals (Cu^+^, Ag^+^, Au^+^) there is, however, also an important contribution of the metal centers to the luminiscence (Scheme 4b) [40,41,45,46,47]. The resulting compounds can be considered thus to be hybrid photoluminiscent d^10^ systems where the emission features can be fine-tuned by introducing either structural variations in the coordination environment of the metal centers or in the ligands. In the present study we aim at expanding the knowledge about the effects of coordination of a flexible organic ligand to a group 11 d^10^ metal ion on the luminescent properties of the resulting polymers. Having in mind the effect of the conformational behavior of the positional isomeric ligand (L1 or L2) and of the d^10^ ion (Cu or Ag), as well as the presence of diverse counteranions when forming the coordination framework-type, we expect to assess if there is any influence of these factors on the luminescent properties in these compounds.

**Figure 7:**
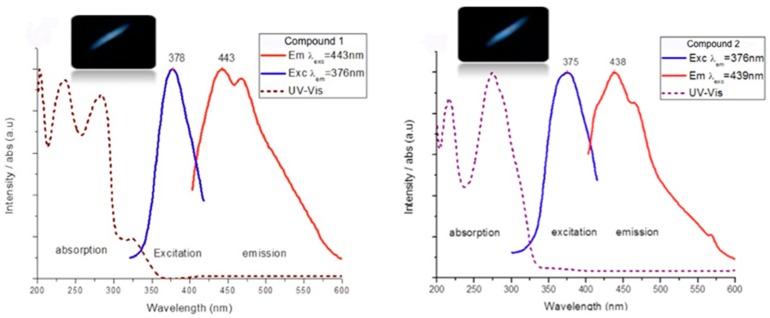

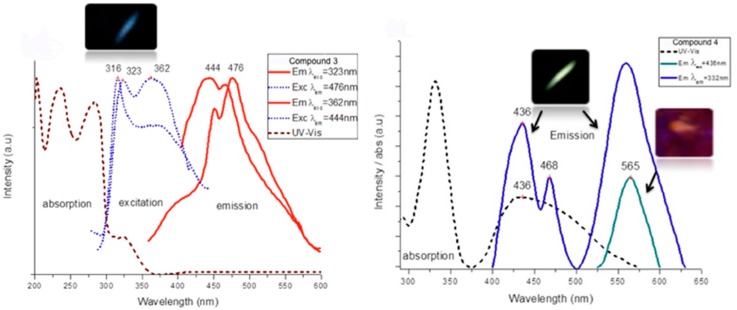
Photoluminescence spectra for compounds **1**–**4** (inset: photography showing the luminescence of each complex under ultraviolet excitation).

To clarify the relative participation of the ligand and the metal ion in the emission, the fluorescence properties of the isolated two ligands, L1 and L2 (Figures S7 and S8, supplementary material), and the four coordination compounds (**1**–**4**) were investigated in solid state samples at room temperature (Figure 7a–e). Both the pure ligands and the metal-organic complexes are strongly luminescent in the solid state at room temperature. The solid-state samples of the pure organic ligands L1 and L2 display a band at 448 nm when excited by irradiation at 383 nm (τ (L2)_439_ = 0.96 ms) and at 403 nm when excited by irradiation at 350 nm (τ(L1)_441_ = 0.93 ms) L1, respectively. The long emission lifetimes seem to support the phosphorescent nature of the luminiscence in the two cases. It should be noted, however, that different conformers are present in the crystals of pure L1 and L2 as verified by X-ray powder diffraction experiments (PXRD) (see Figures S5 and 6 in the supplementary material). While molecules of L1 pack in the crystal structure adopting a TG-type conformation, those of L2, prefer a GG-type one [48,49,50], so that direct comparison of their luminicence properties is not possible.

**Figure 8:**
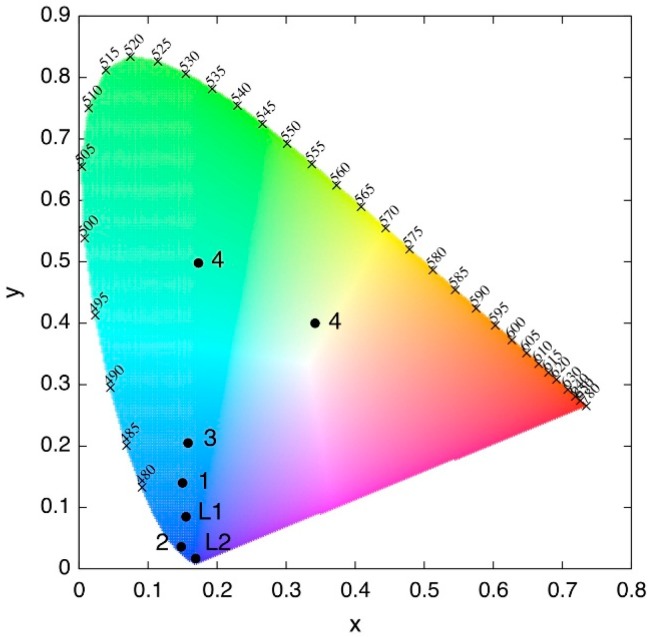
CIE-1932 chromaticity coordinates for pure ligands L1, L2, and compounds **1**–**4**.

Under UV irradiation the photoluminescence of compounds **1**–**4** are distinctly different, indicating that the different network topologies and/or metal ions have a significant influence on the radiative decay processes. Complex **1** shows a high-energy band of emission centered at 443 (τ(2)_443_ = 0.95 ms) and a low-energy band of emission at 467 nm (τ(2)_467_ = 1.20 ms) upon excitation with 378 nm light (Figure 7a) resulting in a bluish emission with CIE chromaticity coordinates (0.150, 0.140) as shown in Figure 8. The emission spectra for **2** (Figure 7b) show a single band at 439 nm with a weak shoulder at ~460 nm (τ(3) = 1.06 ms) upon excitation with a 376 nm source, giving a blue emission with CIE chromaticity coordinates (0.148, 0.036) as shown in Figure 8.

Interestingly compound **3**, exhibits with two distinct emission band profiles (Figure 7c) depending on the excitation wavelength, with emission at 444/457 nm or 451/476 nm (high energy band and low energy band, respectively), when excited by irradiating at 362 or 323 nm (τ(1)_444_ = 1.07, τ(1)_476_ = 1.21 ms) respectively, resulting in an overall bluish emission. The Commission Internationale de l’Eclairage (CIE) chromaticity coordinates [77] for this compound are (0.158, 0.205) as seen in Figure 8. Such changes in the luminescence properties upon excitation wavelength have been previously observed for other silver complexes [78,79].

The emission spectra for **4** (Figure 7d) exhibit strong emission bands at 436 and 565 nm giving a green luminescence with CIE chromaticity coordinates (0.173, 0.498) when excited at 332 nm (Figure 8). However, when excited by irradiation at 436 nm, complex **4** exhibits only the low energy band at 565 nm, changing from a green to yellow emission (CIE chromaticity coordinates: 0.342, 0.400 shown in Figure 8). A dual-mode luminescence behavior is suggested as the origin for the observed color change upon changing the excitation wavelength, although a more thorough computational investigation is being currently performed to confirm this issue.

The long lifetimes, in the *ms* range, found for the emissive states for all four compounds, as well as for the isolated ligands L1 andL2 suggest a triplet origin for the luminescent emission, a situation comparable to that described for other related compounds [45,46]. Details of these results can be found in Table 2.

**Table 2:** Photophysical properties and CIE chromaticity coordinates for pure L1 and L2 and their coordination polymers (**1**–**4**).

Compound	Diffuse Reflectancy λ (nm)	Excitation λ_exc_ (nm)	Emission λ_em_ (nm)	Lifetime τ (ms)	CIE Chromaticity Coordinates
L1	235, 290, 329	349	438	0.96	0.155, 0.085
L2	225, 266, 332	340	407	0.93	0.169, 0.017
{Ag(L1)CF_3_SO_3_}_2_ (**1**)	238, 270, 325	378	443/467	0.95/1.20	0.150, 0.140
{Ag(L1)ClO_4_}_n_ (**2**)	220, 279, 318	376	439	1.06	0.148, 0.036
{Ag(L2)CF_3_SO_3_}_n_ (**3**)	328, 424	323/362	444/476	1.07/1.21	0.158, 0.205
{CuCl(L2)}_n_ (**4**)	332, 436	332 436	436/565 565		0.173, 0.498 0.342, 0.400

In our case, for the complexes of Ag(I) **1**–**3** the red shift in the low-energy emissions as compared to those in the pure ligands is consistent with some silver contribution to the HOMO, so that a triplet metal-to-ligand charge transfer ^3^[MLCT] excited state assignment is tempting. However, the small magnitudes of this red shift suggest that the state involved in luminescence may, instead, be a primarily ligand-based ^3^[LLCT] state that is slightly perturbed by interaction with the silver ions, although the hypothesis of low-energy ^3^[MLCT] emissions in silver complexes seems to be favored in the existing literature [80]. The emissions of complex **2** (Figure 7b) do not have the features of a ^3^[MLCT] transition and we tentatively assign them to ^3^[LLCT] intraligand charge transfer emissions because of the resemblance of their emission energy and lifetime to those observed for the pure L1 ligand.

The emission spectrum for **4** is different to that found for the two above complexes due to the change in the metal ion and its structure type. The emission energy for the metal complex is much lower than that of the free ligand, which leads us to discard an intraligand charge transfer ^3^[ILCT] as its source. Metal to ligand charge transfer ^3^[MLCT] and halide to ligand charge transfer ^3^[XLCT] excited states have been shown to play an important role in the photophysical properties of the copper (I) complexes. Based on these previous examples, the assignment of excited states for **4** must account for major components of chloride-to-ligand charge transfer and metal-to-ligand charge transfer ^3^[(X + M)LCT], (Cl(p_z_) → Lπ*) and (M_3d_ → Lπ*). These suggested assignments are in agreement with published theoretical calculations for Cu(I)-halogen complexes with aromatic heterocyclic ligands [81].

## 4. Conclusions

Two novel metallo-cyclic complexes, {Ag(L2)CF_3_SO_3_}_2_ and {Ag(L2)ClO_4_}_2_ [L2 = propane-1,3-diyl bis-(pyridine-3-carboxylate)], and two novel coordination polymers, {Ag(L1)CF_3_SO_3_}_n_ and {Cu_2_Cl_2_(L1)_2_}_n_ [L1 = propane-1,3-diyl bis-(pyridine-4-carboxylate)], have been synthesized and structurally characterized by X-ray diffraction methods. The luminescent properties in solid-state samples were measured for the pure organic ligands and their complexes, finding that the high-energy emission bands and lifetimes for the complexes are similar to those measured for their respective isolated ligands and, hence, it seems reasonable to attribute these emission bands in the complexes to ^3^[LLCT] intraligand charge transfers. The redshift in the low-energy emissions for compounds **1** and **3** are consistent with the presence of some Ag contribution to the HOMO so that a triplet metal to ligand charge transfer ^3^[MLCT] excited state assignment is tempting. However, the small magnitude of this redshift for compound **2** suggests that the luminescent state in this case may instead be a primarily ligand-based ^3^[LLCT] state, slightly perturbed by interaction with Ag ion. Interestingly compound **4** exhibits dual-mode luminescence with two different emission colors depending on the wavelength of the exciting radiation. A detailed computational study of the excited states for these compounds is actually in progress in our group in order to try to clarify the nature of the observed luminescence in these compounds.

## Supplementary Materials

Supplementary materials can be found at www.mdpi.com/2073-4360/8/2/46/s1.
