# Supplementary material for: Self-Assembly of Discrete Metallocycles versus Coordination Polymers Based on Cu(I) and Ag(I) Ions and Flexible Ligands: Structural Diversification and Luminescent Properties
Source: Polymers (Basel). 2016 Feb 15;8(2):46. doi: 10.3390/polym8020046 (PMC6432529; doi:10.3390/polym8020046)
Supplement: Supplementary file 1 [file polymers-08-00046-s001.pdf]

Supplementary Materials: Self-Assembly of Discrete Metallocycles versus Coordination Polymers Based on Cu(I) and Ag(I) Ions and Flexible Ligands: Structural Diversification and Luminescent Properties

Javier Vallejos, Iván Brito, Alejandro Cárdenas, Michael Bolte, Sergio Conejeros, Pere Alemany and Jaime Llanos

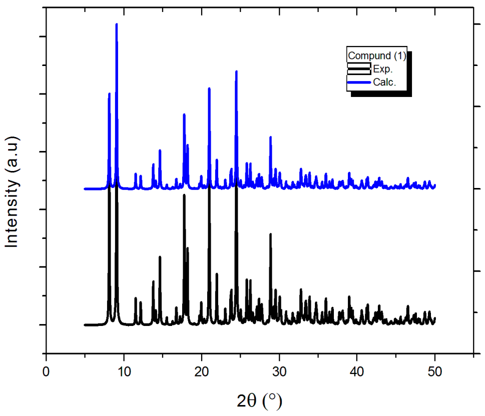

Figure S1. PXRD patterns of complex (**1**).

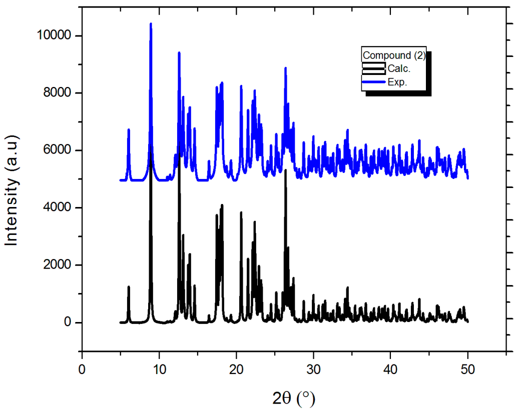

Figure S2. PXRD patterns of complex (**2**).

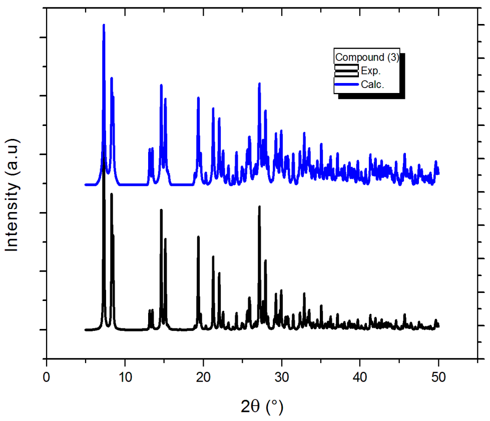

Figure S3. PXRD patterns of complex (**3**).

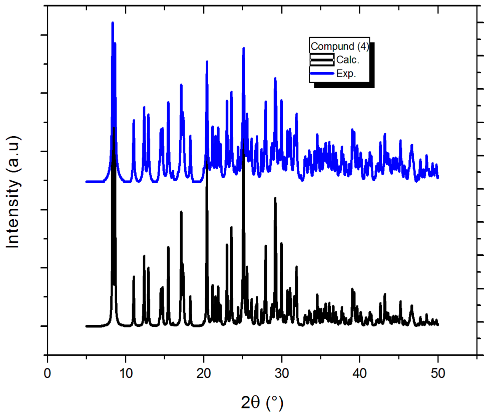

Figure S4. PXRD patterns of complex (**4**).

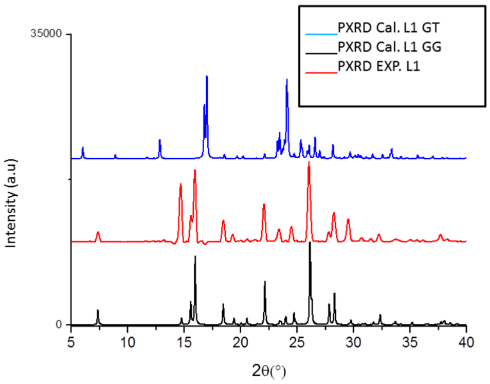

Figure S5. PXRD patterns of L1 bulk.

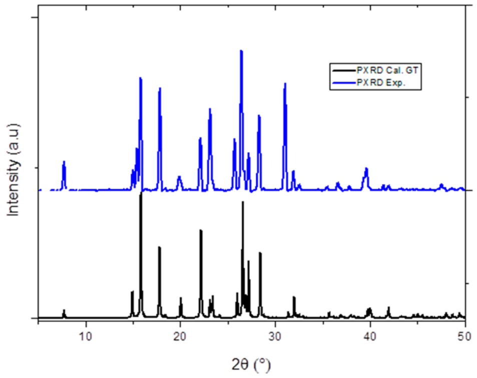

Figure S6. PXRD patterns of L2 bulk.

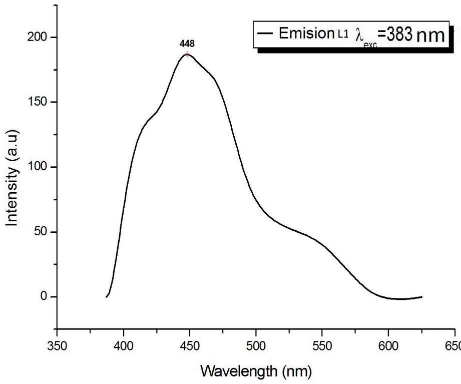

Figure S7. Emission spectra of L1 E_{exc} = 383 nm.

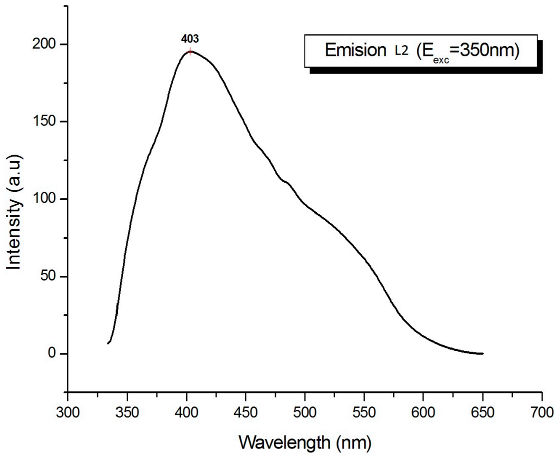

Figure S8. Emission spectra of L2 E_{exc} = 350 nm.

© 2016 by the authors; licensee MDPI, Basel, Switzerland. This article is an open access article distributed under the terms and conditions of the Creative Commons by Attribution (CC-BY) license (http://creativecommons.org/licenses/by/4.0/).
